# Cancer biology and molecular genetics of A_3_ adenosine receptor

**DOI:** 10.1038/s41388-021-02090-z

**Published:** 2021-11-08

**Authors:** Chiara Mazziotta, John Charles Rotondo, Carmen Lanzillotti, Giulia Campione, Fernanda Martini, Mauro Tognon

**Affiliations:** 1 Laboratories of Cell Biology and Molecular Genetics, Section of Experimental Medicine, Department of Medical Sciences, School of Medicine, University of Ferrara, 64/b, Fossato di Mortara Street, 44121 Ferrara, Italy; 2 Center for Studies on Gender Medicine—Department of Medical Sciences, University of Ferrara, 64/b, Fossato di Mortara Street, 44121 Ferrara, Italy; 3 Laboratory for Technologies of Advanced Therapies (LTTA), University of Ferrara, 44121 Ferrara, Italy

**Keywords:** Cancer, Diagnostic markers

## Abstract

A_3_ adenosine receptor (A_3_AR) is a cell membrane protein, which has been found to be overexpressed in a large number of cancer types. This receptor plays an important role in cancer by interacting with adenosine. Specifically, A_3_AR has a dual nature in different pathophysiological conditions, as it is expressed according to tissue type and stimulated by an adenosine dose-dependent manner. A_3_AR activation leads to tumor growth, cell proliferation and survival in some cases, while triggering cytostatic and apoptotic pathways in others. This review aims to describe the most relevant aspects of A_3_AR activation and its ligands whereas it summarizes A_3_AR activities in cancer. Progress in the field of A_3_AR modulators, with a potential therapeutic role in cancer treatment are reported, as well.

## Adenosine and adenosine receptors

### Biology of adenosine

Nucleosides and nucleotides are ubiquitous molecules with several biological functions, which constitute a number of molecule types that are essential for biological processes, including nucleic acids, co-enzymes, energy intermediates and intra-/extra-cellular messengers. The role of both nucleosides and nucleotides, as extracellular messengers, is relevant in some mechanisms, such as cell growth, migration, differentiation, bacterial-induced inflammation and growth factor secretion [1–4]. Adenosine is found at low concentrations (nanomolar range) in physiological conditions, while in stress conditions its concentration increases (micromolar range) [3]. In addition, physiological concentration of circulating adenosine can also vary in different species [5, 6]. In the extra-cellular environment, adenosine partly derives either from ATP, ADP and AMP hydrolysis by specific ectonucleotidases called ectonucleoside triphosphate diphosphohydrolase, or cluster of differentiation 39 (CD39) and ecto-5′-nucleotidase, or cluster of differentiation 73 (CD73) [4, 7]. Intra-cellular adenosine derives from AMP and S-adenosylhomocysteine (SAH) hydrolysis by endo-5′-nucleotidase and SAH hydrolase, respectively. This molecule can either be converted into AMP by adenosine kinase or be deaminated into inosine by the adenosine deaminase enzyme (ADA1 and ADA2) [4]. Adenosine can also be generated through a de novo biosynthesis pathway [8]. In particular, adenosine biosynthesis begins with the generation of ribosyl hypoxanthine monophosphate (IMP), which represents the first purine nucleotide that is synthesized de novo. IMP is initially converted to adenylo-succinate by adenylosuccinate synthase enzyme, while the latter is converted to AMP by adenylosuccinate lyase enzyme [8]. The intra-/extra-cellular concentration of adenosine is mediated by cell membrane complexes, named equilibrative (ENT1–4) and concentrative (CNT1–3) nucleoside transporters [9]. ENTs allow the passive transport of adenosine based on concentration differences, while CNTs use the gradient generated by sodium ions as an energy source to ensure adenosine transport against the concentration gradient [9]. Under physiological conditions, adenosine is transported from the extracellular to the intracellular environment, while, in hypoxic conditions, ENT1 downregulation blocks this flow, thereby leading to an increase in extracellular adenosine [9, 10]. Adenosine is abundant in the tumor microenvironment (TME), which includes tumor-surrounding blood vessels, immune cells, fibroblasts, signalling molecules and the extracellular matrix [11–13]. Adenosine also plays a role in tumor progression, as it is secreted by tumor/immune system cells in TME during this phase [3]. Both tumor promoting and antitumor properties have been reported for adenosine, while high adenosine levels have been reported in TME, as a consequence of hypoxia, which is a typical condition of solid tumors. This increase leads to a pro-angiogenic effect thereby leading to tumor development [3]. Contrariwise, a pro-apoptotic effect for this nucleoside has also been described in leukemia and melanoma in vitro models [3]. The dual role of adenosine seems to also depend on its concentration. Low concentrations (<25 nM) of this nucleoside inhibit tumor growth [14], while high concentrations (100 nM), similar to those determined in TME, confer to adenosine a pro-angiogenic effect [3].

### Adenosine receptors

**Fig. 1:**
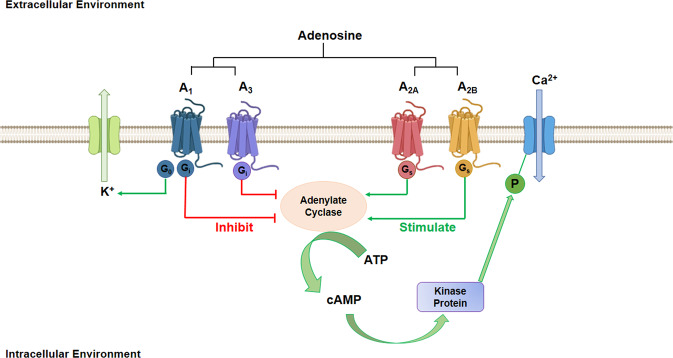
Schematic representation of adenosine receptors. In normal physiological condition adenosine mediates its activation via four G-protein coupled receptors A1, A_2_A, A_2_B, and A_3_. They are primarily associated with activation and inhibition of Adenylate Cyclase. The accumulation of cAMP is linked to the modulation of ion-channel activity.

Adenosine performs its function by binding four different G-protein-coupled adenosine receptors (AR), i.e., A_1_, A_2_A, A_2_B and A_3_ (Fig. 1). The interaction between adenosine and its receptors not only activates pathways involved in different pathological processes, but also promotes receptor expression, in an autocrine manner. ARs comprise a group of glycoproteins with seven transmembrane domains and are coupled to G proteins (Fig. 1) [15]. Adenosine binding to A_1_R/A_3_R causes a decrease in cAMP, while adenosine binding to A_2_AR and A_2_BR causes an increase in cAMP. Evidence indicates that ARs expression appears to be mediated by DNA methylation [16], which is a critical epigenetic process for gene expression regulation [17, 18] in a variety of cell types [19–23].

**Fig. 2:**
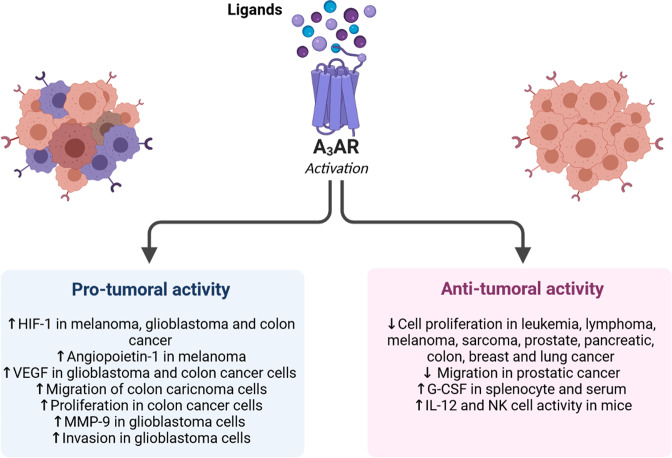
Pro-tumoral and antitumoral activity induced by A_3_ adenosine receptor stimulation in different tumors. The pro-tumoral activity of A_3_AR includes the increase of VEGF, HIF-1, MMP-9, angiogenesis, migration, proliferation, and invasion in tumor cells. The A_3_AR antitumoral activity leads to the decrease of cell proliferation and migration in several tumor cells and the induction of G-CSF and IL-2 secretion from cells of the immune system.

A number of studies have reported that A_3_ receptor (A_3_AR) is overexpressed in cancers [24]. However, the role of A_3_AR in regulating cell proliferation and death is a relatively well debated issue, as this receptor acts differently depending on the tissue type in which it is expressed (Fig. 2) [25, 26]. In vitro models indicate that low (nanomolar) selective synthetic A_3_AR agonists concentrations protect normal cells from death, while A_3_AR agonists present pro-apoptotic effects in both normal and tumor cells at high (micromolar) concentrations. Notably, this dual A_3_AR-ligand mode of action comprises the same signaling pathway following A_3_AR activation (Fig. 2). Based on this evidence, A_3_AR is gaining interest for its potential use as a therapeutic antitumor target [15, 27]. It is thus clear that adenosine and A_3_AR, play a fundamental role in cancer.The aim of this review is to discuss the characteristics of A3AR and its cancer-related activities. Progress in the field of A3AR modulators, with a potential therapeutic role in cancer treatment, will be discussed, too.

## A_3_AR characteristics

### A_3_AR genetic and protein characteristics and tissue distribution

The human A_3_AR coding gene maps on chromosome 1p21-p13. This gene contains two exons separated by a 2.2 kb intron, while encoding a protein of 318 amino acids. The gene regulatory region contains a *cis* sequence, which binds to several transcription factors including NF-κB [28]. The A_3_AR gene promoter upstream region presents a CCAAT consensus sequence and consensus binding sites for SP1, NF-IL6, GATA1, and GATA3 transcription factors [4]. Of these transcription factors, GATA3 is involved in the A_3_AR-dependent role in immune function [4]. A_3_AR protein presents seven alpha-helices containing about 20–27 amino acids. Each helix crosses the cell membrane seven times, while it is connected to an adjacent helix through three intracellular loops and three extracellular loops [29]. The amino-terminal region (N-terminus) is located outside the cell, while the carboxyl-terminal region (C-terminus) is oriented toward the cell cytoplasm. The presence of several tyrosine and serine residues at the C-terminus, confers a desensitization potential on A_3_AR during agonist administration [30]. The phosphorylation of this region leads to a decrease in agonist affinity and an increase in the ability of the agonist to inhibit adenylate cyclase activity, which catalyzes ATP conversion to cAMP [30].

A_3_AR is expressed in enteric neurons, epithelial cells, colon mucosa and lung parenchyma cells, chondrocytes, osteoblasts and also in cells responsible for inflammatory processes, such as mast cells, eosinophils, neutrophils, monocytes, macrophages, dendritic cells, lymphocytes, and bone marrow cells [31, 32]. A_3_AR plays an unclear role in inflammatory processes, as it has been shown to have both anti-inflammatory and pro-inflammatory activites [33, 34]. A_3_AR stimulation on mouse mast cells has been shown to induce degranulation [35]. Conversely, in eosinophils it inhibits chemotaxis, degranulation and generation of superoxide anion [36, 37]. In monocytes and macrophages, A_3_AR inhibits the TNF-α release through the NF-κB signal transduction pathway [38]. In neutrophils, it promotes chemotaxis and inhibits the superoxide anion generation [39]. Furthermore, overexpression of this receptor has been reported in the lungs of patient affected by airway inflammation [40].

### A_3_AR intracellular signaling transduction

A_3_AR can interact with different G proteins, including Gi, Gq, and Go [41]. Specifically, interaction between A_3_AR and Gi protein inhibits adenylate cyclase activity, thus leading to a decrease in cAMP (Fig. 1) [41]. This process causes protein kinase A (PKA) inhibition, which leads to a glycogen synthase kinase-3β (GSK-3β) increase, a β-catenin and cyclin D1 down-regulation, as well as NF-κB-DNA binding potential reduction [41]. Furthermore, A_3_AR regulates the mitogen-activated protein kinase (MAPK), phosphatidylinositol 3-kinase (PI3K)/Akt and NF-κB signaling pathways [29]. It is known that PKA/PKB/Atk phosphorylate and inactivate GSK-3β, which is a key element of the Wnt signal. In its active form, GSK-3β suppresses cell proliferation [42]. When A_3_AR is activated, a decrease in cAMP levels occurs thereby leading to a reduction in the phosphorylated PKB/Akt and PKA active form [42]. This phenomenon causes a dysregulation in the Wnt signal transduction pathway, which increases cell proliferation and, thus, tumorigenesis [28]. When A_3_AR activates G proteins, phospholipase C activity is stimulated with a consequent increase in calcium concentration and protein kinase C (PKC) stimulation. This process induces TNF-α release in active macrophages [4].

## The role of A_3_AR in cancer

### General overwiew

Cancer can be a deadly disease, which is caused by alterations in gene expression and pathways [43, 44]. A number of useful diagnostic/prognostic markers have been identified for numerous tumor types [44, 45]. A_3_AR is overexpressed in cancer and it is considered a tumor diagnostic/prognostic marker, as previous studies have demonstrated its overexpression in different malignant tumors including melanoma, breast, prostate, liver, pancreatic and lung cancers, as well as lymphoma, glioblastoma and malignant pleural mesothelioma (MPM) [3, 29, 46, 47]. High levels of A_3_AR in cancer cells and blood cells have also been demonstrated in colorectal cancer patients [47, 48].

A_3_AR stimulation is known to inhibit tumor growth by regulating the Wnt pathway [49]. GSK-3β plays a key role in this molecular process, as it is responsible for β-catenin phosphorylation. When phosphorylated, β-catenin induces the transcription of genes, which are fundamental for the cell cycle progression, such as c-myc and cyclin D1. By treating cancer cells with A_3_AR agonists, GSK-3β levels increase, while cyclin D1 and c-myc expression is suppressed. This molecular effect, which induces a decrease in cancer cell proliferation [28], has been found in melanoma, hepatocellular carcinoma, as well as in the synoviocytes of patients suffering from rheumatoid arthritis [4].

The dual nature of A_3_AR in cancer has been remarked upon (Fig. 2). In some tumors it promotes cell proliferation and survival, while in others it triggers cytostatic and apoptotic pathways [28]. A_3_AR stimulation inhibits lung cancer proliferation by arresting the cell cycle [50]. A similar effect has also been observed in vitro in murine lymphoma [51]. Adenosine-dependent A_3_AR stimulation induces apoptosis in stomach cancer cells via a mechanism that involves PKC activation [52]. An inhibition of tumor growth following receptor stimulation has been demonstrated in lymphoma [51], leukemia, [53] as well as colon [54] and pancreatic carcinoma [55]. Contrariwise, A_3_AR stimulation prompts cell proliferation in other cancer types, such as colorectal cancer and adenocarcinoma [56]. In human glioblastoma cells, A_3_ARs stimulation induces an increase in MMP-9 following ERK, PKA/Akt activation, causing an increase in cell invasiveness [46, 52, 57].

### Hypoxia and A_3_AR regulation

**Fig. 3:**
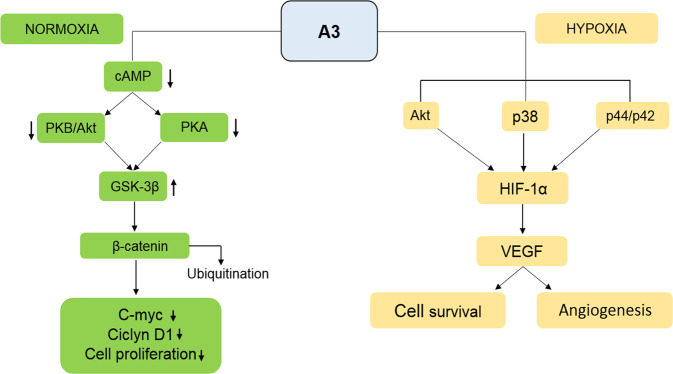
A_3_ receptor pathways in normoxia/hypoxia conditions. Cellular pathways activated by the stimulation of the A_3_ receptor in normoxia and hypoxia conditions.

Hypoxia is a common phenomenon in many solid tumors [58]. In hypoxia conditions, where there is a lack of O_2_, adenosine accumulates in TME, while factors involved in cellular response to hypoxia, such as *hypoxia inducible factor 1 (HIF-1)* are released [59]. HIF-1 is abundant in tumor cells surrounded by TME, where it plays a fundamental role in angiogenesis, invasion as well as in the alteration of tumor cell metabolism [60, 61]. HIF-1 is a heterodimer protein made up of an α and a β subunits, which are HIF-1α and HIF-1β. Although both subunits are constitutively expressed, the expression level of α subunit increases when O_2_ concentrations decrease [62]. Since both HIF-1 and A_3_AR have been found to be overexpressed in cancer, a link between A_3_AR stimulation and the modulation of HIF-1α expression in hypoxic conditions has been explored [59, 63]. In some tumor types, such as melanoma, glioblastoma and colon cancer, HIF-1α expression has been found to be increased by adenosine-induced Atk and/or MAPK signal pathway activation [59]. Adenosine involvement in the production of *vascular endothelial growth factor (VEGF)* in cancer has also been investigated. In vitro evidence indicates that in glioblastoma cell lines, A_3_AR activation stimulates *VEGF* expression [64]. Another study has reported that treating colon cancer cell lines with A_3_AR antagonists blocks both *HIF-1α* and *VEGF* expression in hypoxic conditions [12]. A similar effect has also been observed in melanoma cell lines, where adenosine stimulated A_3_AR causing *HIF-1α* over-expression in response to hypoxia [59]. To demonstrate that the HIF-1α increase depends on A_3_AR stimulation, whereas it is not due to other ARs, antagonists from other AR family members, including A_1_R, A_2_AR, A_2_BR, were used. The positive effect of adenosine in increasing HIF-1α was only determined following A_3_AR stimulation. In the same study, p44/p42 and p38 MAPK were demonstrated as necessary in order to increase HIF-1α levels (Fig. 3) [59].

Additional data support a correlation between A_3_AR and MAPK/ERK pathway [65]. A_3_AR-mediated ERK activation has been reported in human fetal astrocytes, microglia and several tumors, such as colon carcinoma, glioblastoma, and melanoma [59, 66–68]. On the contrary, ERK inhibition leads to a reduction in cell proliferation in melanoma, prostate cancer and glioma [67, 68]. Different in vitro models, such as hamster ovary cells [69], melanoma [59], colon carcinoma and glioblastoma [56, 70], indicated that A_3_AR is also responsible for p38 MAPK activation, while the opposite result has been reported in synoviocyte cultures [71]. A_3_AR is also responsible for activating C-Jun N-terminal kinase patway in microglia and glioblastoma cells [4]. As a result, this mechanism causes an increase in cell migration and *matrix metallopeptidase-9 (MMP-9*) overexpression [4].

**Fig. 4:**
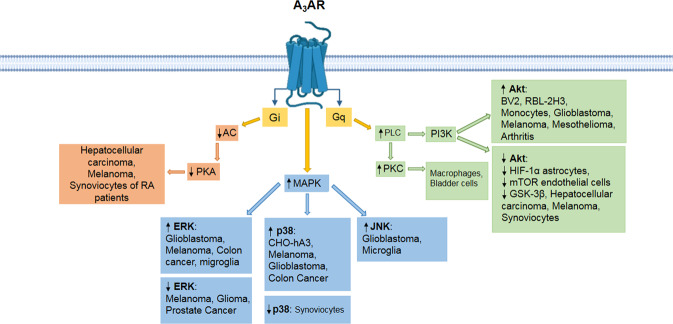
A_3_ receptor pathways. Representation of signal transduction pathways induced by the stimulation of the A3 receptor.

A_3_AR stimulation causes Akt phosphorylation [65]. In glioblastoma cells and in mouse basophilic cancer cells Akt phosphorylation causes apoptosis inhibition [70, 72], while the same pathway demonstrates an anti-proliferative effect in human melanoma cells [73]. A_3_AR also mediates PI3K/Akt signal activation [65]. The PI3K/Akt and NF-κB signal transduction pathways are mediators of the anti-inflammatory effect, which has been observed in BV-2 microglial cells [74], monocytes and mesothelioma cells [46, 75]. PI3K/Akt and NF-κB pathway inhibition reduces HIF-α and GSK-3β concentration (Fig. 4) [4, 25].

Animal models indicate that adenosine plays a key role in regulating vascularization in melanoma [76]. Specific activation of A_1_AR, A_2_AR and A_3_AR in CD73-knockout mice affected melanoma growth, neovascularization, angiogenesis, and macrophage infiltration. Specifically, A_3_AR activation induced pro-angiogenic factor expression and secretion by mast cells, macrophages infiltration and cytokines expression in TME via a paracrine mechanism [76]. In another study conducted on a human melanoma cell line, it has been reported that A_3_AR stimulation can induce a reduction in cell proliferation [73]. Indeed, after receptor stimulation, the PI3K/Akt signal transduction pathway is activated, leading to a reduction in ERK1/2 [73]. This molecular effect ultimately inhibits cell proliferation [73].

## A_3_AR agonists and antagonists in cancer treatment

### General overview

**Fig. 5:**
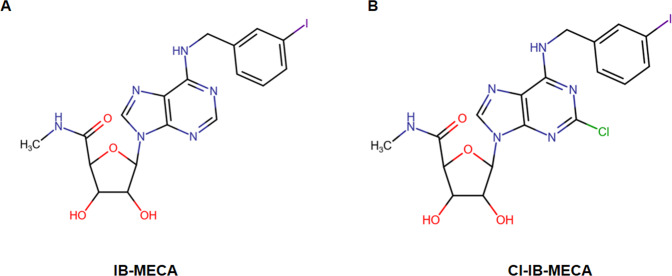
A_3_ receptor ligands. Chemical structures of (**A**) N6-(3-Iodobenzyl)adenosine-5′-N-methyluronamide (IB- MECA; CF101); **B** 2-chloro-N6-(3- iodobenzyl)-adenosine-5′-N-methyluronamide (Cl-IB-MECA; CF102).

A_3_AR offers a promising therapeutic target for inflammatory diseases, such as rheumatoid arthritis and psoriasis [77]. Its importance in treating cancer is also increasing [78]. Numerous agonists, partial agonists, allosteric modulators and antagonists have been developed [79]. The main drugs employed in clinical trials are N6-(3-Iodobenzyl)-adenosine-5′-N-methyluronamide (IB-MECA; CF101) and 2-chloro-N6-(3-iodobenzyl)-adenosine-5′-N-methyluronamide (Cl-IB-MECA; CF102) (Fig. 5). These molecules, which have shown positive results in preclinical studies, are considered safe and have been well tolerated during clinical trials [80–83]. IB-MECA and Cl-IB-MECA derive from adenosine and contain a lipophilic substituent (3-iodobenzyl) in position N6 and modified ribose in position 5′ [84]. Cl-IB-MECA contains a further substituent, chlorine, which makes it more selective than IB-MECA (Fig. 5). The first developed selective agonist IB-MECA is 50 times more selective for A_3_AR than other ARs [84]. However, in addition to affinity, other parameters must also be considered, such as the half-life of the agonist, the duration of the response induced by the receptor link and efficacy in vivo. Additional molecules, derived from other nucleosides, act as partial agonists or antagonists [4]. Both have been shown to be safe and effective. Currently, IB-MECA is being tested in phase I clinical trial for psoriasis (NCT00428974) and phase II and III clinical trials for rheumatoid arthritis (NCT01034306 and NCT02647762), while Cl-IB-MECA is being tested in phase I and II clinical trials for liver cancer (NCT00790218 and NCT02128958) and in the treatment of non-alcoholic steatohepatitis (NASH, NCT02927314). Both IB-MECA and Cl-IB-MECA, which are administered orally, are safe and well tolerated.

In vivo experiments indicate that A_3_AR is not desensitized following chronic treatment [85]. Other data indicate that shortly after its last administration, A_3_AR down-regulation occurs, while 24 h after the last administration of the drug, expression levels of the receptor return to those shown by the control group. Therefore, chronic treatment does not reduce A_3_AR expression [85].

The antitumor effect of A_3_AR agonists occurs via Wnt/NF-κB pathway modulation [34]. PKB/Akt, NF-κB and TNF-α expression levels have been shown to be reduced during treatment with A_3_AR agonists in both in vitro/in vivo liver carcinoma models [86]. Based on reported data, synthetic A_3_AR agonists induce apoptosis and inhibit cell proliferation in different types of cancer cells in in vitro/in vivo models. These drugs are safe and well tolerated, mainly due to their cardio- and neuro-protective effects and therefore, they can be considered as new therapeutic approaches to some cancer types.

### A_3_AR agonist: IB-MECA

The antitumor effects of IB-MECA have been associated with GSK-3β up-regulation and NF-κB, cyclin D and c-Myc down-regulation [87]. Furthermore, colon cancer animal models have indicated that A_3_AR activation via IB-MECA interaction inhibits tumor growth. Indeed, in tumor tissues taken from IB-MECA-treated mice, a decrease in the level of PKA expression and an increase in GSK-3β, which led to the down-regulation of β-catenin, has been observed [54]. Subsequent in vitro studies revealed that this effect is hampered by administrating GSK-3β inhibitors, which confirms the involvement of A_3_AR [54]. IB-MECA induces down-regulation of the PKB/Akt signal transduction pathway, consequently inhibiting NF-κB activity in vivo/in vitro [54]. IB-MECA also prevents the formation of liver metastases from colon cancer cells inoculated into the spleen [25]. In addition, IB-MECA significantly blocks breast cancer cell motility and metastasis [88]. IB-MECA is also responsible for prostate cancer cell proliferation suppression. At low concentrations IB-MECA arrests the cell cycle in phase G1, while at a high concentrations it induces apoptosis by increasing the activity of pro-apoptotic proteins caspase-3 (CASP3) and Bax, while Bcl-2 expression levels decrease [89]. However, IB-MECA also blocks cell cycle progression in phase G1 at concentrations between 0.001 and 10 µM in ovarian cancer cell lines, where it also causes a decrease in cyclin D1 and cyclin-dependent kinase 4 levels [90].

### A_3_AR agonist: Cl-IB-MECA

Cl-IB-MECA pharmacokinetic parameters and long-term safety have been analyzed in a study involving hepatocellular carcinoma patients. Cl-IB-MECA-induced apoptosis has been reported to occur by modulating the Wnt signal transduction pathway, while showing a protective effect on healthy cells. These promising results suggest that this compound could also be used on patients with other diseases, such as liver cirrhosis or inflammation [91]. Cl-IB-MECA is able to inhibit lung metastases formation in mice with melanoma at a nanomolar concentration range [27]. In addition, Cl-IB-MECA shows a synergistic antitumor effect when employed in combination with cyclophosphamide [27]. A study conducted on MPM indicates a key role for Cl-IB-MECA as an anticancer agent [46]. This neoplasm is strongly correlated to asbestos exposure. Evidence also suggests the involvement of Simian Virus 40, which is an oncogenic virus member of the *Polyomaviridae* family [92, 93], as an MPM co-factor [94]. During MPM onset, macrophages secrete TNF-α, which activates pro-inflammatory pathways, leading to NF-κB activation, thereby promoting survival. In inflammatory conditions, adenosine concentrations increases, as A_3_AR have been shown to be involved in the NF-κB/Akt pathway. When tumor cells are treated with Cl-IB-MECA, the level of Akt phosphorylation decreases [42, 65]. Cl-IB-MECA is able to inhibit the effect of TNF-α on the survival/proliferation of cells exposed to asbestos, by inhibiting NF-κB activation [46]. This treatment induces lactate dehydrogenase and CASP3 release in MPM cells, implying a cytotoxic effect mediated by A_3_AR [46]. These findings suggest that A_3_AR could represent a target for preventing MPM development.

Cl-IB-MECA can be effective against other cancer types. Data from lung cancer cell lines experiments indicated that 0.01–10 mM of both adenosine and Cl-IB-MECA induces apoptosis in a dose-dependent manner [42]. A study conducted on thyroid carcinoma reported that Cl-IB-MECA inhibits tumor growth and blocks cell cycle progression in tumor cells [95]. This effect is related to a reduction in cyclin D1 expression and the dephosphorylation of ERK1/2, which both depend on treatment time and drug concentration. In addition, in vivo Cl-IB-MECA potentiates the activity of NK cells by inducing IL-12 production, a cytotoxic factor with antitumor effects [57].

A_3_AR has been found overexpressed in tumor cells and peripheral blood mononuclear cells in patients with hepatocellular carcinoma [48]. Treatment with Cl-IB-MECA inhibits the growth of hepatocellular carcinoma cells in orthopic mouse models in a dose-dependent manner. Moreover, the effect of Cl-IB-MECA is due to NF-κB down-regulation and an increase in GSK-3β [86]. Induced apoptosis also depends on an increase in the expression of pro-apoptotic proteins, such as Bad, BAX and casapase-3 [86].

### A_3_AR agonist: Thio-Cl-IB-MECA

Using agonists as antitumor agents, such as 2-chloro-N6-(3-iodobenzyl)−4′-thioadenosine-5′-N-methyluronamide (thio-Cl-IB-MECA), has also brought encouraging results. Thio-Cl-IB-MECA is able to inhibit the cell cycle progression in lung cancer cells [50]. It can also induce apoptosis at a high concentrations via cyclin D1, c-Myc and cyclin-dependent kinase 4 (CDK4) downregulation as well as CASP3 and −9 activation [50]. Thio-Cl-IB-MECA induces apoptosis in human leukemic cells and has an anti-inflammatory effect, as it inhibits the expression of pro-inflammatory cytokines by modulating PKB/Akt pathways and NF-κB [50, 57, 96].

### A_3_AR antagonists

Results on receptor stimulation support the hypothesis that receptor antagonists may also be useful in treating different cancer types [97]. Animal models have confirmed that activating the receptor in melanoma cells induces an increase in blood vessel density, pro-angiogenic molecule secretion, cytokine production and the invasion of macrophages into the tumor [4]. In glioblastoma, A_3_AR stimulation prompts MMP-9 expression with a consequent increase in cell invasiveness, while using antagonists co-adiuvates the antitumor effect of chemotherapy [70]. These results have been obtained following in vitro studies, but studies conducted on animal models are needed to support these data and confirm the idea that antagonists may also represent a valid therapeutic approach [4, 29]. A3AR antagonists employed in clinical trials include PBF-1650 (NCT03798236) phase I for Psoriasis and PBF-677 (NCT02639975, Glaucoma, phase I) (NCT03773952, Ulcerative Colitis, phase II).

## Concluding remarks and future perspectives

In conclusion, the studies reported in this review indicate A_3_AR and its ligands as key players in cancer onset/development. Previous in vitro/in vivo data indicate that A_3_AR has been found as overexpressed in a variety of cancer types, including glioblastoma/glioma, colon, breast, and pancreatic cancers, as well as MPM and lymphoma. A_3_AR could thus potentially be used as a tumor diagnostic/prognostic marker and target for anticancer therapy. A_3_AR has become an attractive therapeutic antitumor target, as its agonists show encouraging results in preclinical studies. Furthermore, some of those agonists are also currently being tested in clinical trials, while A_3_AR antagonists have also obtained encouraging results in preclinical studies.

Research focused on the role of A_3_AR and its modulators in cancer is an essential future area of study. As A_3_AR appears to be enigmatic in terms of its effects, investigating the dual nature of this receptor is an important research field, which deserves attention. Although a growing number of studies have investigated the structure and function of A_3_AR, its regulative role on modulating both cell proliferation and cell death by interacting with its large variety of ligands, represents a relatively well debated issue due to its dual role depending on a tissue specific context and on ligands type/concentration [26]. On this ground, further studies focusing on the dual nature of A_3_AR activation, as well as the potential interaction of these two opposing responses to tumor growth, are needed. Since A_3_AR stimulation activates a number of pathways, the study of these mechanisms could be essential for understanding the role of these receptors in cancer onset/progression and metastasis. Further in vitro/in vivo studies into A_3_AR mechanisms upon cancer are, thus, also to be encouraged. Novel data could improve cancer diagnosis, the prognostic management of cancer patients, as well as the development of novel therapies.
